# The selection advantages associated with advanced biological maturation vary according to playing position in national-level youth soccer

**DOI:** 10.5114/biolsport.2023.119983

**Published:** 2022-10-06

**Authors:** Liam Sweeney, Sean P Cumming, Áine MacNamara, Dan Horan

**Affiliations:** 1 School of Health and Human Performance, Faculty of Science and Health, Dublin City University, Dublin, Ireland; 2 Department for Health, University of Bath, Bath, United Kingdom; 3 High Performance Department, Football Association of Ireland, Dublin, Ireland

**Keywords:** Biological maturation, Talent development, Football Association of Ireland, Association football, Soccer

## Abstract

This study investigated the extent to which biological maturation selection biases existed according to playing position in national-level youth soccer. A total of 159 players from the U13 to U16 age groups in the Football Association of Ireland’s national talent pathway and international representative squads had their relative biological maturity status assessed using the Khamis-Roche method for the percentage of predicted adult height at the time of observation. Players were categorised as goalkeeper (GK), central defender (CD), full-back (FB), centre defensive midfielder (CDM), centre midfielder (CM), centre attacking midfielder (CAM), wide midfielder (WM) or centre forward (CF). A series of one-sampled means t-tests were used to examine the degree to which biological maturation selection biases existed across playing positions. A non-parametric Kruskal-Wallis test was used to evaluate inter-positional differences. A small to very large selection bias in favour of early maturing players existed for GK (D = 0.7), CD (D = 1.65), FB (D = 0.49), CM (D = 0.62), WM (D = 0.78), and CF (D = 0.76) (p < 0.05). Maturational selection biases did not exist for CDM or CAM. Moreover, CD were significantly more advanced in maturation compared to FB, CDM and CAM (p < 0.05). This study supports the contention that maturation selection biases exist in youth soccer, but the magnitude of this bias is highly dependent upon playing position. The very strong maturity selection biases at the national level evidenced in this investigation highlight the need for Football Associations to explore strategies, such as futures programmes, to help to retain talented, yet late maturing athletes.

## INTRODUCTION

Biological maturation is the process of progression toward the mature adult state and can be defined in terms of status, timing and tempo [1, 2]. Maturation status describes the stage of maturation an individual has attained at the time of observation (i.e., prepubertal, pubertal, postpubertal); whereas timing refers to the chronological age at which specific maturation events (e.g., peak height velocity, menarche) occur [2]. Tempo refers to the rate at which maturation progresses [1]. Biological maturation is governed predominantly by a combination of genetic and, to a lesser extent, environmental and behavioural factors (i.e., chronic malnutrition, disease, climate) [3]. Human tissues, organs and organ systems mature; however, this occurs independent of chronological age [3]. Thus, youth of the same chronological age can vary substantially in their status, timing and tempo of maturation [4]. For instance, from late childhood, same age peers have been shown to vary by as much as five-to-six years in skeletal age, an established index of maturation status in youth [5, 6]. The individual differences in biological growth and maturation are central to the identification and development of talented youth soccer (football) players [7].

Early maturing males are generally taller and have greater muscle mass, fat mass and muscular strength compared to their on-time and later maturing counterparts [8]. As a direct consequence, early maturing youth outperform later maturing youth in tests of muscular strength, power and speed [9, 10, 11]. Advanced skeletal maturity is also associated with higher absolute VO_2_ peak values in adolescent footballers [12]. In a youth football context, these physiological and functional advantages transfer directly into match-play, with players advanced in maturation performing more high-intensity actions, covering more high-speed distances, and achieving faster peak speeds than later maturing players [13, 14]. These strength and performance advantages increase the likelihood of the early maturing boys being perceived as more talented at the point of selection and being recruited into the development system [7].

The selection bias toward players advanced in maturation emerges at the onset of puberty (circa 11–12 years) and increases in magnitude with chronological age and the level of competition [15, 16]. The disproportionate overrepresentation of early maturing boys within professional football academies is well documented [4, 15, 16, 17], and early maturing boys have been shown to constitute as much as 74% of players within some academy cohorts [16]. As a direct consequence, late maturing boys are underrepresented within professional football academies, and in some instances, are deselected by age 14–15 years [15]. Indeed, early maturing boys are up to twenty times more likely to be retained within the academy system [16]. From a developmental perspective, many equally talented yet late maturing boys are subsequently denied exposure to the professional coaching, sports science and medical support, superior training equipment and facilities, and the high levels of competitive challenge that are experienced by those within the academy system [15, 16]. Once excluded, these late maturing players are less likely to be represented at the professional level [15].

Although the selection of youth players more advanced in maturation may elicit some immediate advantages (i.e., competitive success), it can be counterproductive in regard to long-term player development. The physical, physiological and functional advantages associated with advanced biological maturation are attenuated or even reversed by adulthood [4]. Consequently, by excluding later maturing players, football clubs and associations have excluded a large proportion of the available pool of talent to select from, in turn reducing the number of high potential players that can be produced for the senior level of the game. Of interest, several scholars have proposed that later maturing players may hold the greatest potential for senior success [4, 18]. The reasoning behind this proposition is attributed to the late maturing players being forced to overcome their biological limits through the development of other skills necessary for successful performance, including superior technical skills and tactical understanding, as well as the utilisation of more adaptive engagement in self-regulated learning, particularly self-evaluation and learning [4, 18]. The high-pressured academy environment may also encourage early maturing boys to utilise their athletic advantages in order to be successful at the expense of their technical-tactical and psychological development [4]. Moreover, early maturing players are likely underchallenged when competing against less mature players who are not as physically able, which may further hinder the development of the technical-tactical and psychological skills necessary to succeed at the highest levels [19, 20].

Despite our increasing understanding of the role of biological maturation on talent identification and development in youth football, there is a scarcity of research that has investigated how variations in maturation differ between players of different playing positions. It would seem logical to presume that the techno-tactical and physical requirements of specific playing positions influence how players at different stages of biological maturation are perceived by coaches. Malina et al. [21] investigated variations in skeletal maturation in national-level youth footballers by playing position and found that forwards were the most advanced, followed by defenders and then midfielders. However, the participant sample was small (n = 17) and aged only between 15–16 years, and players were broadly categorised as either defender, midfielder or forward (goalkeepers excluded). In contrast, using the same positional categorisations, other authors have found differences in skeletal maturation between regional-level youth players to be negligible [22]. Regardless, these positional categorisations fail to recognise the distinct physical, technical and tactical variations between players of the same broad playing position (e.g., full-backs vs. central defenders, central defensive midfielders vs. central attacking midfielders). More research is required to understand how variance in biological maturation differs between specific playing positions in youth footballers using larger participant samples, more specific positional categorisations, and a broader range of age groups. Investigating how maturation biases vary according to playing position would help to further support the talent identification and development process by allowing clubs and coaches to identify in which positions the physical and physiological advantages associated with advanced maturation are most influential. Moreover, findings would reveal whether coaches have a perception of the physical characteristics that they think players in specific positions should have at the point of selection, rather than looking at final attributes. These findings may help to support the provision of effective strategies to help to retain talented, yet later maturing players. With consideration to the previous discussion, the purpose of this investigation was to examine the variations in biological maturation between different playing positions in national-level youth footballers.

## MATERIALS AND METHODS

### Participants

159 players enrolled in the Football Association of Ireland’s (FAI) national talent pathway at the time of investigation had their estimated relative biological maturation status assessed (FAI national talent development programmes begin at the U13 age group). The players assessed were either U13, U15 or U16 national-level players. Data was collected before training sessions over the first quarter of the year and each player was assessed once. 100% of players selected into a given national squad that were invited to participate in this research consented, making the sample representative of all players within the national pathway in the cohorts under investigation. The sample consisted predominantly of European Caucasians. Each parent/guardian and player received an information leaflet outlining the purpose of the research study and provided written informed consent before data collection. Ethical approval was granted by the first author’s institutional Research Ethics Committee and conformed to the ethical standards of the Helsinki Declaration.

### Biological maturity status

The maturity status of each player was estimated using the percentage of predicted adult height [23, 24]. Among children of the same chronological age, it is assumed that those closer to their predicted adult height are more advanced in maturation compared to those further removed from their predicted adult height. The Khamis-Roche method enables the prediction of a player’s adult height using the regression formula based upon age and gender-specific regression coefficients detailed by Khamis and Roche in their analysis of residents enrolled in the Fels longitudinal study [23, 24]. The Khamis-Roche protocol requires the current age, height and weight of the child, and biological mid-parent height (mean height of biological parents). Players had their body height measured to the closest 0.1 cm using a stadiometer (SECA, 217, Vogel and Halke, Hamburg, Germany) and their body mass measured to the closest 0.1 kg using digital scales (SECA, 877, Vogel and Halke, Hamburg, Germany). Parents’ heights were self-reported and then adjusted for overestimation as outlined by Epstein et al. [25].

Mean adjusted paternal and maternal heights (178 cm and 165 cm, respectively) were in line with sex-specific means for Irish adults [26]. In instances where a biological parent was not in contact with a player and their parent/guardians, a national average for adult height was used for that biological parent (this accounted for approximately 8% of the sample). The median error bounds between actual and predicted adult height using the Khamis-Roche method is 2.2 cm in males aged between 4 to 17.5 years. For the age groups examined in this study, 12 to 16 years, the lowest 50% error was 1.3 cm for 16-year-olds, and the highest 50% error was 2.8 cm for 14-year-olds [23, 24]. Using the percentage of predicted adult height has demonstrated construct validity as an indicator of biological maturation status in samples of healthy American, Portuguese and German youth [17, 27, 28].

The height of each player was expressed as a percentage of predicted adult height which was used as an estimate of biological maturity status at the time of observation [29]. Estimated relative biological maturity status was then expressed as a Z-score using the child’s percentage of adult height compared to age-specific means and standard deviations outlined by Bayer and Bailey in the Berkeley Growth Longitudinal Study [30]. Comparisons to reference values outlined by Bayer and Bailey have been utilised in recent football-specific studies on other European players [7, 15, 17, 19, 31]. These Z-scores were then used to classify the youth players as late, on-time or early maturing. A Z-score of -0.5 to + 0.5 was classified as on-time maturity status; a Z-score of > + 0.5 was classified as early maturity status; and a Z-score of < -0.5 was classified as late maturity status (as currently employed in the English Premier League Player Management Application; [15]). The ± 0.5 criteria were selected over the traditional ± 1 criteria to better differentiate between players that were more advanced or delayed. Using the latter criteria, players presenting maturity Z-scores of -.99 and + .99 would be both considered ‘on-time’, when in reality there is almost 2 standard deviations of variance in maturation between them. All growth and maturation assessments and calculations were made by the first author who was trained using standardised field practices.

### Playing position

Players were categorised as either goalkeepers (n = 17), full-backs (n = 22), central defenders (n = 20), central defensive midfielders (n = 17), central midfielders (n = 30), central attacking midfielders (n = 12), wide midfielders (n = 23), and centre forwards (n = 18). Indeed, whilst recognising that youth players of such ages (12–16 years) are encouraged to be flexible and perform in several playing positions and tactical formations, for the purpose of this investigation, corroboration took place with each players’ national head coaches in which the players’ “best” position was chosen, which was subsequently assigned to the player for the analysis.

### Data Analysis

Data were analysed using SPSS Version 27. Descriptive statistics were used to examine the variance in biological maturation status relative to chronological age (Z-Scores) across playing positions. Given the unequal sample sizes across different playing positions, a non-parametric Kruskal-Wallis test was used to evaluate group differences. Post-hoc comparisons were determined by the Bonferroni technique (0.05 was used for statistical significance). A series of one-sample means t-tests (one-tailed) were also used to examine the degree to which relative biological maturation selection biases existed across each playing position by comparing the observed mean values for relative biological maturation (Z-Score) against the values expected for the general population (0.0). Subsequent tests of equivalence were used to determine the magnitude of any biases and the degree to which any biases were or were not equivalent to the absence of bias. A 90% confidence interval that existed within the ± 0.5 Cohens D equivalence band was accepted as equivalent to the absence of bias. In contrast, confidence intervals that crossed the ± 0.5 Cohens D equivalence band were accepted as not equivalent to an absence of a bias. Effect sizes (Cohens D) were used to examine the magnitude of any significant differences (small = 0.2–0.49; moderate = 0.5–0.79; large = 0.8–1.19; very large = ≥ 1.2) [32].

## RESULTS

**FIG. 1:**
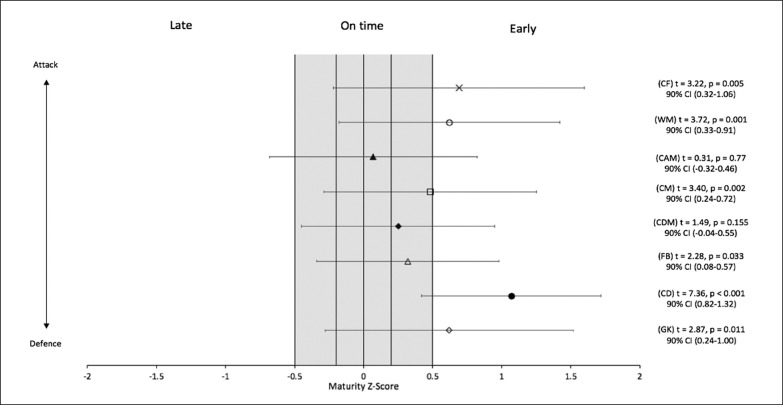
Maturity selection biases by playing position from the series of one-sampled means t-tests. Data presented as Mean ± SD maturity Z-score for each position. GK = Goalkeeper FB = Full-back CM = Central midfielder WM = Wide midfielder CD = Central defender CDM = Central defensive midfielder CAM = Central attacking midfielder CF = Centre forward

**FIG. 2:**
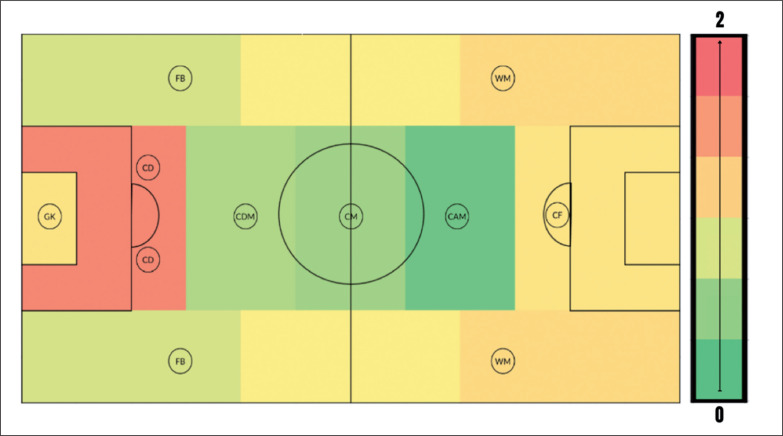
Cohens D effect sizes for the mean values for relative maturation status (Z-Score) by playing position on the soccer field, attacking from left to right. Note the green value denotes a lower effect size and thus a lower selection bias in favour of early maturation, whereas red denotes a higher effect size and a larger selection bias. The scale denoting the effect size ranges (Cohens D) is displayed to the right. GK = Goalkeeper FB = Full-back CM = Central midfielder WM = Wide midfielder CD = Central defender CDM = Central defensive midfielder CAM = Central attacking midfielder CF = Centre forward

The results of the one-sample mean t-tests to examine the presence of maturity selection biases by playing position are presented in Table 1. The mean value for relative maturation was significantly greater than the expected value for goalkeepers, central defenders, full-backs, centre midfielders, wide midfielders, and centre forwards (p < 0.05) (Figure 1). The magnitude of the statistically significant maturation biases ranged from small to very large (Cohens D = 0.49–1.65) (Figure 2). Each significant maturation bias was considered not equivalent to the absence of bias in the one-sampled mean t-tests. The result of the Kruskal Wallis analysis indicated that there was a significant difference in relative maturation status across playing positions (H (7) = 19.31, p = 0.007). Subsequent pairwise comparisons showed that central defenders were significantly more mature for their age than centre attacking midfielders (p = 0.011), defensive midfielders (p = 0.027) and full-backs (p = 0.019). There were no other significant differences between positions.

**TABLE 1:** Descriptive statistics (Mean ± SD) for relative biological maturity status in the Irish football player pathway by playing position. Note the expected values for maturity Z-Scores for the general population are 0.0.

Playing position	n	Maturity Z-Score Mean ± SD	Cohens D (90% CI)	Magnitude of the selection bias
Goalkeeper	17	0.62 ± 0.90^*^	0.70 (0.24–1.13)	Moderate
Central defender	20	1.07 ± 0.65^*^	1.65 (1.06–2.20)	Very large
Full-Back	22	0.32 ± 0.66^*†^	0.49 (0.11–0.85)	Small
Central defensive midfielder	17	0.25 ± 0.70^†^	0.36 (-0.06–0.77)	No significant difference
Central midfielder	30	0.48 ± 0.77^*^	0.62 (0.29–0.94)	Moderate
Central attacking midfielder	12	0.07 ± 0.75^†^	0.09 (-0.34–0.56)	No significant difference
Wide midfielder	23	0.62 ± 0.80^*^	0.78 (0.38–1.16)	Moderate
Centre forward	18	0.69 ± 0.91^*^	0.76 (0.31–1.19)	Moderate

Total sample	159	0.54 ± 0.80^*^	0.67 (0.53–0.81)	Moderate

* Denotes a significant difference between the observed value and expected value.

† Denotes a value significantly less than the value observed for centre defenders

## DISCUSSION

The purpose of this study was to investigate variations in biological maturation in national-level youth footballers of different playing positions. When taking the mean values for relative maturation status by position, a selection bias in favour of earlier maturation existed for goalkeepers, central defenders, full-backs, central midfielders, wide midfielders and centre forwards with effect sizes ranging from small to very large. In contrast, biological maturation selection biases were not observed for central defensive midfielders or central attacking midfielders. Controlling for differences in sample size, central defenders were significantly more mature than central attacking midfielders, central defensive midfielders and full-backs, with no other significant differences observed between positions.

Although there is a lack of existing studies to directly compare to the current investigation, Malina et al. [21] investigated variations in maturation status according to outfield playing position, noting forwards and defenders to be more mature than midfielders, with forwards being the most advanced. These findings were not replicated in the present study. Central defenders were the most biologically advanced, with a Z-Score notably higher than those observed for centre forwards (1.07 vs. 0.69). Central defenders were also significantly more advanced in maturation than two of the three midfield playing positions (central defensive and central attacking midfielders). Importantly, direct comparisons to the findings of Malina et al. [21] are done so with caution given the differences in sample sizes examined (17 vs. 159) and the number of different playing positions (3 vs. 8) analysed. It is equally important to note that the current sample represented a very select group of players that had been identified as the most able within a specific nation and, as such, greater selection pressures or a particular preference for style of play may have influenced the nature of the results. In line with the findings of this study, defenders have also been observed as the most mature players within a similar sized sample of French academy players, although differentiation between full-backs and central defenders was also not conducted [33].

At the youth level, players advanced in maturation perform more high-intensity actions (running ≥ 1 s at > 19 km · h^−1^), repeated highintensity actions, and attain faster peak speeds during matchplay [13]. In addition, early maturing boys are generally taller and have greater muscle mass and strength [8] which is beneficial during physical challenges. It is thus unsurprising that the maturation biases are more pronounced in the outfield positions in which many of these physical characteristics are most desirable. Central defenders were the most biologically advanced in this sample. Indeed, defenders were also amongst the most mature players in similar samples [21, 33]. This is likely due to the technical-tactical positional requirements in which greater size/stature and strength (e.g., to compete in aerial duals) is highly desirable in this position. During elitelevel match-play, sprinting is the most frequent action in goal-scoring situations [34]. As advanced maturation is associated with increased levels of testosterone and maturation of the anaerobic system [3, 35], this may explain why the centre forwards and wide midfielders (the most offensive positions) selected are amongst the most mature players. Central defenders, centre forwards and wide midfielders had mean Z-Scores between 0.62–1.07, demonstrating a moderate to very large maturation bias (Cohens D = 0.76–1.65). As well as being taller and having greater muscle mass and strength, early maturing boys are generally heavier and have greater fat mass [8, 13]. As body size dimension is proposed as one of the most important prerequisites to becoming a professional goalkeeper, this may explain the extent of the maturation bias observed for goalkeepers [36]. The selection biases in favour of early maturing players within professional football academies are well documented [4, 15, 16, 17]. The magnitude of this selection bias emerges at the onset of puberty and increases with chronological age and the level of competition [15, 16]. Results from this study demonstrate that the magnitude of these selection biases is also heavily influenced by playing position. It has previously been suggested that maturation biases may be the most prevalent in the central positions [15], but these results do not directly support this hypothesis. Interestingly, wide midfielders and full-backs were generally early maturing and more biologically advanced than both central attacking and defensive midfielders.

During senior-level match-play, central attacking midfielders have been shown to compete in the lowest number of aerial duals but spend the most time in possession of the ball and have the highest number of ball possessions with a role focussed on linking midfield and attack [37]. Likewise, central defensive midfielders have a role focussed on building passing sequences, with less emphasis on sprinting and high-speed running metrics compared to the other midfield/offensive positions [37, 38]. Given that the physical and physiological advantages associated with early maturation (e.g., increased lean muscle mass, power, strength, anaerobic metabolism) are not primary technical-tactical components of central attacking and defensive midfielders, this may explain why early maturation biases are not observed in these positions. However, it is important to note that the extent of the total, high-intensity and sprinting distances performed by players of each position during match-play is highly variable (e.g., influenced by formation, score-line, minute of match-play). Additionally, a small selection bias in favour of early maturing players was observed in full-backs, although full-backs were significantly less mature than central defenders. This may be attributed to the fact that full-backs operate in less congested areas of the pitch where such physical characteristics associated with advanced maturation may be less desirable. Typically, young players are identified and then selected based on subjective analysis by coaches on the factors thought to underpin senior performance; coaches may have a perception of the physical characteristics that they think players in specific positions should have. Although the physical, physiological and functional advantages associated with early biological maturation do not influence the (de)selection of central defensive midfielders or central attacking midfielders at the national level in this sample, this does not imply that late biological maturation does not influence the deselection of players in these positions in this sample.

### Practical implications

In this sample of national-level youth footballers, selection biases in favour of early maturation were shown to exist in some, but not all, playing positions. Nevertheless, mean Z-scores for each position were positive values (≥ 0.07). These results suggest that late maturation does appear to be a negative selection factor in all positions, especially for central defenders and centre forwards. By preferentially selecting early maturing players for specific playing positions, a large proportion of the available pool of talent to select from is reduced, and in turn, so is the potential to maximise the quality and quantity of players produced for the senior level of the game [16]. Given that all players mature by adulthood, and therefore senior levels of performance, the effects of advanced maturation will no longer be present by this point. Yet, the later maturing boys deselected from the system are unlikely to return in the future despite possibly holding potential for senior success [4, 16, 18]. Late maturing youth who are techno-tactically and psychologically skilled need to be nurtured through the system until biological maturity is attained [33]. However, talent identification is a difficult and complex process for coaches. Late biologically maturing players may likely be ineffective performers in match-play during their adolescent and early teenage years in comparison to their early maturing peers, making it challenging for coaches to identify attributes in these players that suggest that they have the potential to develop into outstanding senior level performers. Contrastingly, even the best performing adolescent footballers often fail to attain elite-level senior status demonstrating that the road to senior success is a rocky one [39] and a road that many players may be excluded from travelling upon.

To maximise the opportunity for late maturing players to be retained within the national system, Belgium started a Futures Programme in 2008 [40]. This Futures team has consisted of players that have been identified as technically, tactically, and psychologically able, yet delayed in physical maturity. These teams compete against other nations and academy teams, enabling opportunity for late developers to be retained within the system and experience what it is like to compete and travel as part of a national team. Further research should examine the degree to which Future’s teams are successful in retaining late maturing players and allowing them to succeed at the highest level. Indeed, the Belgian Futures programme has been in place for 14 years, and there are case examples of successful outcomes, although there is no published data to support its effectiveness in long-term player development, highlighting the bio-psychosocial complexity of talent development.

In immediate terms, several other strategies could be used to support the development of late maturing players, as well as players of on-time and early maturation statuses. One potential intervention is to reduce the pitch sizes during training/competition to emphasise the use of technical skills over physical/physiological attributes (and advantages) [41]. Similarly, matching players by maturity status during one-on-one training drills or matches (e.g., playing a right wide midfielder against a biologically matched left back) may help to support this process. In broader terms, the provision of generic coach education workshops and resources that educate clubs and their coaches in the area of growth and maturation may be a supplementary level of support that could be provided by Football Associations. Ongoing educational support for practitioners at the club and national level in the appropriate monitoring and training of the adolescent athlete would allow staff to support individual players based upon their physical needs.

### Limitations

Whilst recognising that dividing players by eight playing positions rather than three reduces the sample sizes within each position, we believe our positional categorisations provide a more sensitive measure to examine maturation given the large variations in the technical-tactical (e.g., aerial duals, forward passes, crosses) and physical (e.g., high-speed running distance, sprint distance, number of accelerations and decelerations) demands between players in different positions (e.g., full back vs. central defender, attacking midfielders vs. defensive midfielder). Indeed, the overall sample size within this study is larger than similar studies conducted previously [21, 22, 33]. This investigation was conducted with national-level youth footballers and provides a contextualised assessment of maturation biases according to playing positions in a real-world setting. Second, parental heights for the prediction of adult height were self-reported and adjusted for overestimation using equations based upon participant samples from the United States [25]. The percentage of predicted adult height at the time of observation was used as the indicator of biological maturity status using the regression formula and coefficients outlined by Khamis and Roche, which was derived from samples of American youth of European ancestry [23, 24]. The Z-scores used to derive maturity status from the percentage of predicted adult height are calculated based on participants of European ancestry [30].

## CONCLUSIONS

This study investigated the variations in biological maturation between different playing positions in national-level youth footballers. The findings indicated that central defenders were significantly more mature than full-backs and central defensive and attacking midfielders. Moreover, selection biases in favour of advanced maturation existed for goalkeepers, central defenders, full-backs, central midfielders, wide midfielders and centre forwards, although the magnitude of this bias ranged from small to very large. Selection biases did not exist for central defensive and attacking midfielders. This study supports the contention that maturation selection biases exist in youth football, but the magnitude of this bias is dependent upon playing position.
